# Single breast incision for breast conservation surgery and sentinel lymph node biopsy: a systematic review

**DOI:** 10.1111/ans.19389

**Published:** 2025-01-31

**Authors:** Lucy P. Aitchison, Andrew J. Spillane

**Affiliations:** ^1^ Breast and Melanoma Surgery Department Royal North Shore Hospital Reserve Rd St Leonards New South Wales Australia; ^2^ MIA Translational Hub The University of Sydney Wollstonecraft New South Wales Australia; ^3^ Breast and Endocrine Surgery Department Mater Hospital Wollstonecraft New South Wales Australia

**Keywords:** breast conserving surgery, minimal access breast surgery, minimally invasive breast surgery, sentinel lymph node biopsy, single incision

## Abstract

**Background:**

Breast conservation surgery and sentinel lymph node biopsy via a single incision allows excision of tumours from all quadrants of the breast, with access to both axillary and internal mammary nodal basins with no additional incisions.

**Objectives:**

This systematic review aims to consolidate the current literature on the efficacy, safety, functional and cosmetic outcomes of single‐incision breast conserving surgery and sentinel lymph node biopsy.

**Data sources and review methods:**

A comprehensive search of Pubmed, EMBASE, Medline and GoogleScholar was conducted from inception to 7th July 2024 for all peer‐reviewed articles assessing breast conserving surgery and sentinel lymph node biopsy via single incision using PRISMA guidelines.

**Results:**

The literature search generated 426 articles. 400 were excluded by abstract review with the remaining 26 articles reviewed in full. An additional three articles were retrieved from review of full article reference lists. 13 articles were excluded, leaving 10 articles meeting the inclusion criteria. The technique demonstrated success across all quadrants of the breast. Eight studies documented successful axillary dissection completed via a single incision. Four studies compared the single‐incision approach to the conventional two‐incision technique. There was no difference in complication rates. The single‐incision technique was associated with higher patient satisfaction and reduced post‐operative axillary pain.

**Conclusion:**

Single‐incision breast conservation surgery and sentinel lymph node biopsy is surgically safe and can be feasible for tumours in all quadrants of the breast and is associated with improvement in cosmesis, pain and patient satisfaction. Further studies are required to confirm its long‐term oncological safety.

## Background

Single‐incision breast conserving surgery (BCS) and sentinel lymph node biopsy (SLNB) allows for wide local excision of the tumour and access to both the axilla and internal mammary nodes via a single breast incision. It has previously been termed MABS (minimal access breast surgery)^1^ and TANE (transmammary axillary lymph node evaluation).^2^ The technique was first described in 1994 by Smellie *et al*. from the Royal Marsden Hospital, UK, during a period where there was a movement towards more aesthetically aware oncological choices with the use of Kraissl rather than Langer's lines^3^ as well as the start of oncoplastic breast surgical practice.^4^

The breast incision is chosen without compromising the best cosmetic and oncological outcomes for the breast. BCS is completed prior to SLNB. Access to the draining lymph node basin is achieved via mobilization along the chest wall between layers of the pectoral fascia using good assistance, adequate lighting and long, thin retractors (as depicted in Figure 3).^1^ Once the lateral edge of pectoralis major is reached via the retromammary space, the clavipectoral fascia is opened to access the axillary nodes.^5^ Bromberg and Giordano^6^ use the assistance of an extra small Alexis wound retractor (Applied Alexis wound retractor, Applied Medical Resources Co., Rancho Santa Margarita, CA, USA) to make benefit of the ‘moving breast window’.^7^ This process is easier if the breast is larger, particularly if it is also ptotic but can often be achieve across a broad range of breast phenotypes.

**Objectives:** This review consolidates the current literature on the efficacy, safety, functional and aesthetic outcomes of single‐incision breast conserving surgery and sentinel lymph node biopsy.

## Methods

**Fig. 1:**
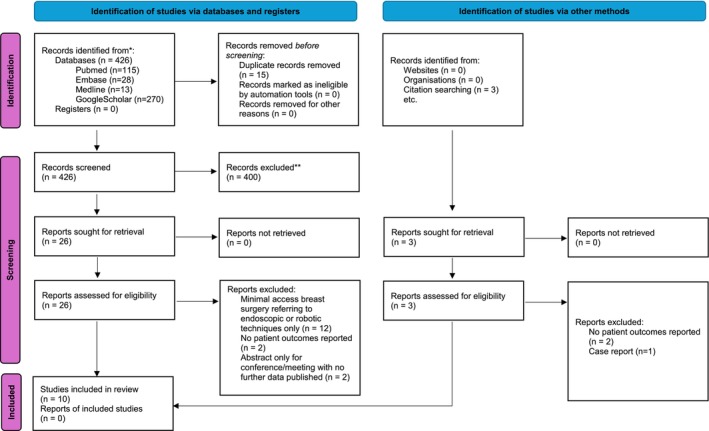
PRISMA 2020 flow diagram. *Source*: Adapted from: Reference 8 .

A comprehensive search of EMBASE, Medline, Pubmed, and GoogleScholar was conducted from inception to 7 July 2024 using PRISMA guidelines for peer‐reviewed articles assessing single‐incision BCS for the treatment of breast cancer and access to the axilla.^8^ This included studies that assessed oncoplastic techniques as part of BCS, as well as studies that included axillary dissection (AD) as well as SLNB. The references of the included articles were also reviewed to identify additional relevant publications. See Appendix S1 for the full search strategy used and Figure 1 for the PRISMA flow diagram.

Studies were excluded if ‘minimal access breast surgery’ or ‘minimally invasive breast surgery’ referred to endoscopic‐assisted, or robot‐assisted breast surgery. Case reports and surgical ‘how I do it’ descriptive studies with no patient outcomes where were excluded.

The outcomes assessed included the success rate for removal of sentinel nodes, the number of nodes harvested, patient and surgeon satisfaction, axillary pain, cosmesis, complication rate, oncological outcomes and surgical time.

## Results

The literature search generated 426 articles. Four hundred were excluded by abstract review with the remaining 26 articles reviewed in full. An additional three articles were retrieved from review of full article reference lists. Thirteen articles were excluded, leaving 10 articles meeting the inclusion criteria. Reasons for exclusion included ‘minimal access breast surgery’ referring to endoscopic‐assisted or robotic‐assisted techniques only, if no patient outcomes were reported or if the article was a case report.

A total of 10 studies were identified that met the inclusion criteria. Four studies compared a single‐incision approach for BCS and SLNB or AC versus a conventional two‐incision technique. Three of these were retrospective^9^, ^10^, ^11^ and one of these was prospective.^5^ The remaining six studies were observational only with no comparison group. The number of patients, success of technique (axillary and internal mammary nodal basins), number of nodes removed, surgical time and complication rate are summarized in Table 1.

**Table 1:** Summary of included studies and reported outcomes in their single‐incision arm

Study first author (year published)	Number of patients in single‐incision arm	Success of single incision technique for axilla nodal harvest (%)	Success of single incision technique for IM and axillary SLNB (%)	Average number of nodes removed	Surgical time (minutes)	Complication (%)
Cocilovo (2006)	44	97.73	NR	Mean 2.74 (SD NR)	NR	0
Spillane (2009)	100	86	55	Mean 2.82 (SD 1.47)	NR	1
Bromberg (2016)	31	100	NR	NR	NR	NR
Bromberg (2018)	34	100	NR	Median 2 (IQR 1–5)	Median 132 (IQR 1.8–2.8)	0
Lovasik (2018)	48	100	NR	Median 2 (IQR 2–3)	Median 56 (IQR 46.5, 73.3)	20
Acea‐Nebril (2019)	152	98.7	NR	Mean 2.14 (SD 0.99)	Mean 88.54 (SD 49.35)	7.9
Bromberg (2020)	94	100	NR	Mean 3.6 (SD 4.1)	Mean 160 (SD NR)	0
Nguyen‐Strauli (2022)	86	97.7	NR	Mean 2.03 (SD 1.31)	Mean 91 (SD 33)	8
Morsy (2022)	30	100	NR	NR (range 5–29)	NR	20
Mohsen (2023)	20	100	NR	NR	NR	15

IQR, interquartile range; NR, not reported; SD, standard deviation.

### Tumour location

**Fig. 2:**
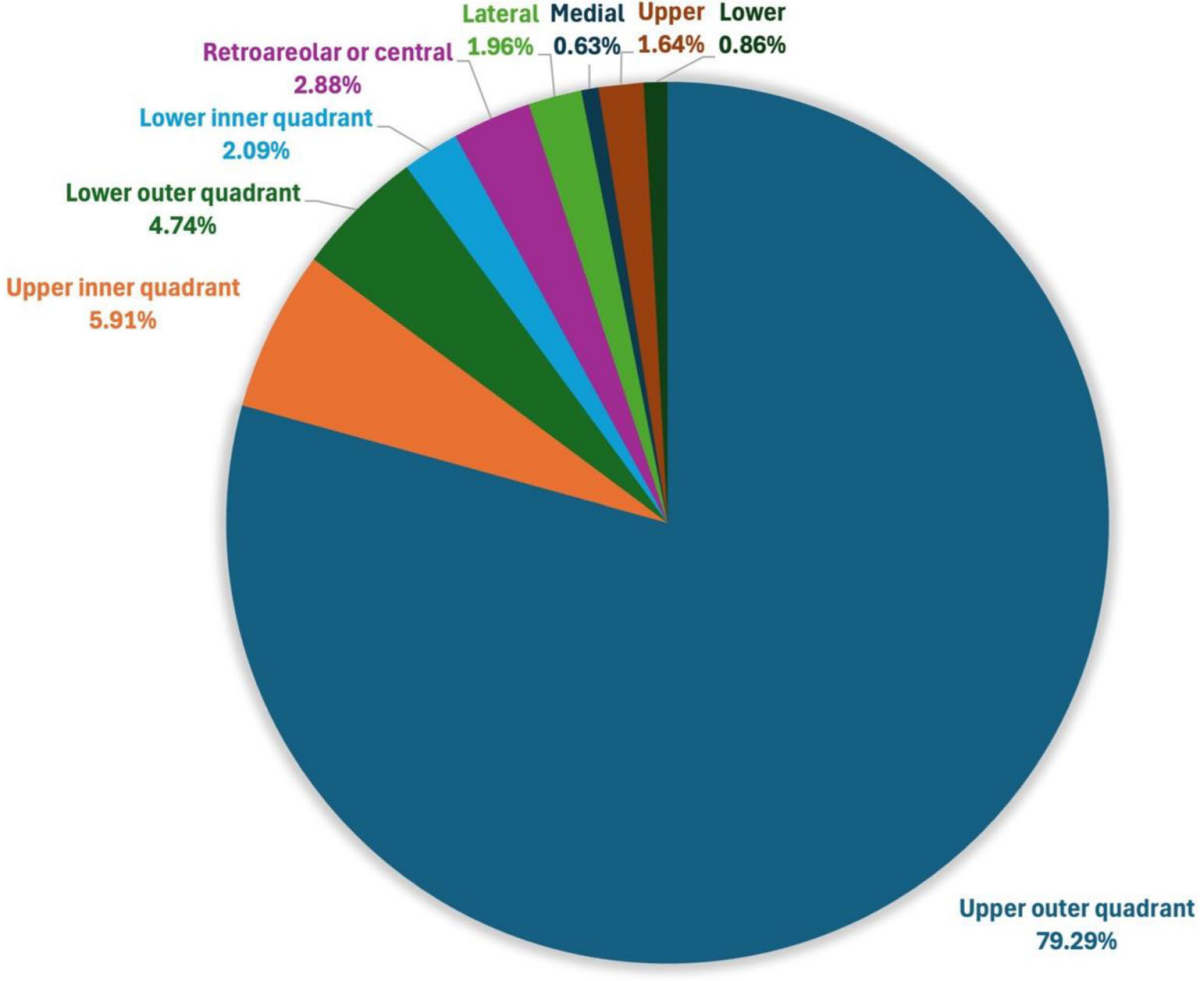
Distribution of tumours (single‐incision approach only).

The distribution of tumour location is summarized in Table S2 and Figure 2. Across all studies, 79.29% of tumours were located in the upper outer quadrant. In their axillary SLNB only group, Spillane and Brennan^1^ reported an overall success rate of 86%, but a 96% success rate if the tumour was in the upper outer quadrant or lateral, compared with 50% where the tumour was located medially, inferiorly or in the lower inner quadrant.^1^ Acea‐Nebril *et al*.^5^ had two of 154 patients that required conversion of the procedure to an additional axillary approach. For both these cases, the incision was from the inframammary fold for tumours in the upper inner quadrant.

**Fig. 3:**
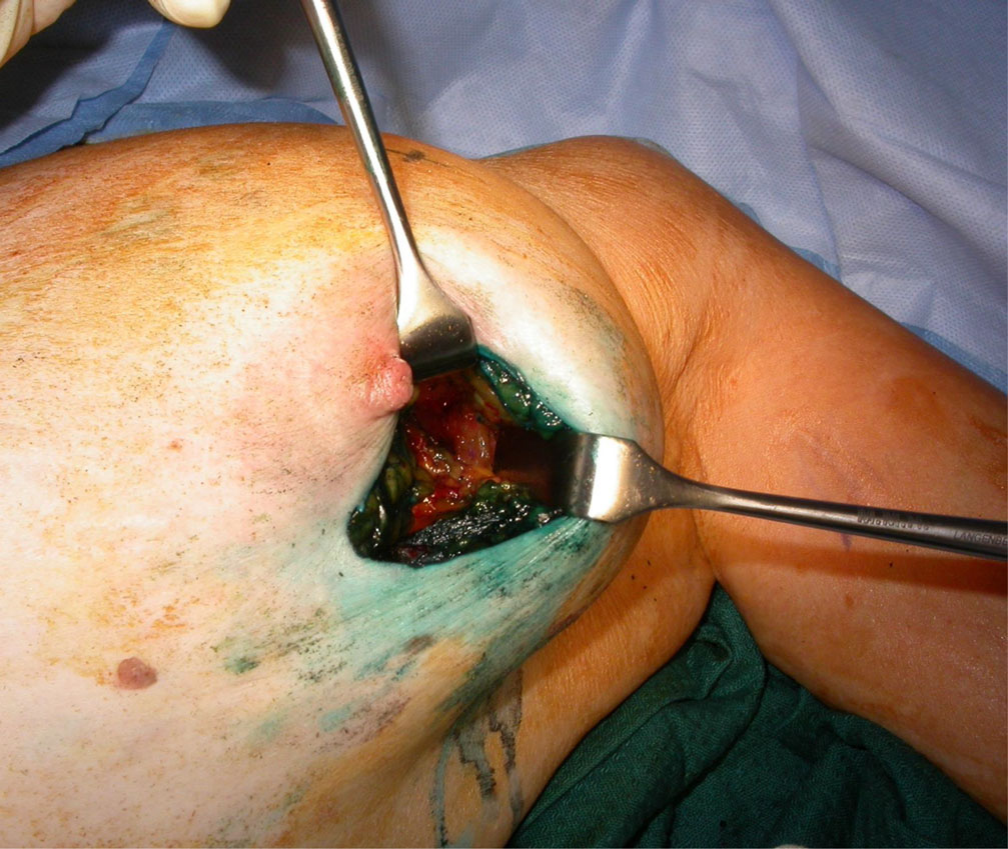
Intraoperative photograph of the single incision technique with access to the axilla via a periareolar incision. Long thin retractors (10 cm in this case) are used to achieve adequate exposure.

### Nodal harvest

The average number of dissected nodes was reported in eight studies with all studies reporting a mean or medial between 2 and 4 nodes.^1^, ^2^, ^5^, ^6^, ^9^, ^10^, ^11^, ^12^ Acea Nebril^5^ found no difference in number of sentinel lymph nodes excised between their single incision versus standard two incision groups (mean 2.14 ± 0.99 vs. 2.04 ± 1.09, *P* = 0.284), nor in the number of positive sentinel lymph nodes (0.35 ± 0.65 vs. 0.36 ± 0.75, *P* = 0686).

AD via a single incision was assessed in eight studies.^1^, ^2^, ^5^, ^6^, ^9^, ^10^, ^13^, ^14^ All 17 patients who underwent AD in the case series by Mohsen *et al*.^13^ had tumours in the upper outer quadrant with incision in the lateral mammary fold. Spillane and Brennan^1^ reported nine patients who had AD via the breast incision. Seven tumours were in the upper outer quadrant, with one in the upper inner quadrant and one in the lower outer quadrant. The mean number of nodes dissected in 15 cases of AD via a separate incision was 13 (range 6–23) compared with 19.4 (range 8–31) in the single‐incision group. Of the nine AD performed in the study by Cocilovo *et al*. four patients required a separate axillary incision. The number of nodes removed via a single incision ranged from 5 to 17, versus 10 to 30 in the patients that required a separate incision to be made.^2^ Similarly, Bromberg *et al*. had a trend towards increased number of nodes excised in the two‐incision group (median 4, IQR: 1 to 13) compared with (median 2, IQR: 1 to 5) in their single‐incision arm although these results encompass a combination of both AD and SLNB with no further distinction or reporting of the percentage of AC patients in each arm.^9^

Dissection of the internal mammary nodes (IMN) via a single breast incision was only reported by Spillane and Brennan.^1^ Seventeen patients underwent BCS and axillary and internal mammary SLNB via the same incision and 13 patients had a single incision for BCS and internal mammary SLNB with a separate axillary incision for axillary SLNB. One patient required three separate incisions to safely access the breast and both lymph node groups, however, the presence of a breast prosthesis in this case was likely a confounding factor. This was the only patient in their study that required a parasternal incision to access the IMN.^1^

### Cosmesis

Cosmesis was not formally assessed by any study with a validated scoring system. For tumours in the upper outer quadrant, Mohsen *et al*.^13^ report excellent aesthetic outcomes in 80% of cases – rated by the patient and two surgeons with consideration of volume symmetry, shape of breast contour, symmetry of nipple‐areolar complex (NAC) position, ipsilateral scarring and post‐irradiation consequences. Nguyen‐Strauli *et al*.^11^ found increased axillary skin retraction in the two‐incision arm in their retrospective cohort series of 134 patients (6.3% vs. 0, *P* = 0.019).

### Patient satisfaction

Acea‐Nebril^5^ assessed patient satisfaction with the Breast‐Q™ questionnaire and found that the single incision group had higher post‐operative satisfaction with both the breast (mean of 78.17 ± 15.97 vs. 66.65 ± 13.76, *P* = 0.002) and the information provided by the surgeon (85.32 ± 16.59 vs. 72.10 ± 20.99, *P* = 0.01). There was no difference found in other aspects of the Breast‐Q™ questionnaire including physical psychological or sexual wellbeing at 12–24 months post‐completion of adjuvant radiotherapy.^5^

### Pain

Nguyen‐Strauli *et al*.^11^ defined axillary neuralgia as pain in the armpit present at three or more months post‐operatively and found increased axillary neuralgia in their two‐incision arm (10.4% vs. 1.2%, *P* = 0.0139). Acea‐Nebril *et al*.^5^ also reported a trend towards increased axillary neuralgia in their two‐incision arm (5.41%) compared with their single‐incision arm (1.32%) although this did not reach statistical significance with their cohort of 226 patients.

### Surgical time

Three studies found decreased surgical time with the single‐incision group including Lovasik *et al*.^10^ (median of 56 min (IQR 46.5, 73.3) vs. 64 min (IQR 54.5, 75.5), p 0.026), Nguyen‐Strauli *et al*.^11^ (mean of 91 min ± 33 vs. 113 min ± 37 *P* < 0.001) and Bromberg *et al*.^9^ (median 130 min (range 30–220) vs. 180 min (range 50–275)). The extended surgical times reported by Bromberg *et al*. are likely as a result of the additional use of intra‐operative radiotherapy as well as frozen section in their patients.^9^

Conversely, Acea‐Nebril *et al*.^5^ found that the single‐incision group had longer surgical time (88.54 ± 49.35 min vs. 69.93 ± 38.43, *P* = 0.017). However, when divided into lumpectomy versus use of oncoplastic technique (41% of cases in the single‐incision arm vs. 18% in the two‐incision arm), no difference in time was found. We also note that the patients who had level two oncoplastic techniques performed in this study also had symmetrisation performed on the contralateral breast and this was included in the surgical time.^5^

### Complication rate

The most common complications reported in the majority of studies included breast haematoma, seroma, wound infection or dehiscence, skin retraction and nipple necrosis.

No statistically significant difference in complication rate was demonstrated in any of the comparative studies between a single‐incision versus conventional two‐incision.^9^, ^10^, ^11^

While not statistically significant, Nguyen‐Strauli *et al*.^11^ reported a trend towards a difference in location of seroma between the two groups, with the single‐incision group having more breast seromas (7% vs. 0%) whereas the two incision group had more axillary seromas (8% vs. 1%). Acea‐Nebril *et al*.^5^ also assessed lymphoedema rates and found no difference between the two groups (2.70 vs. 2.63% *P* = 0.208).

### Oncological safety

Bromberg *et al*.^9^ assessed margins and local recurrence – they reported adequate margins with no need for reoperation or recurrence in either arm, although their cohort was small (60 patients in total), with a median follow‐up time of only 7.5 months (range 1–16 months). This was despite a decreased excision volume in the single incision arm (16.3 (2–90) vs. 42.4 (5–270)). None of the studies reported long‐term oncological data.

## Discussion

This review has summarized the efficacy and safety of the current published literature regarding single‐incision BCS and SLNB. Whilst cosmesis was only formally reported by a single study,^13^ the aesthetic benefits of a single‐incision technique are inherently evident. Not only does it avoid the additional scar, but the retromammary dissection allows for better glandular approximation to provide excellent cosmetic outcomes in both breast contour and skin. This is supported by increased patient satisfaction formally assessed by Acea‐Nebril *et al*. in their Breast‐Q™ questionnaire although it should be noted that their results may be confounded by the high proportion of patients who underwent level I and II oncoplastic procedures (compared with lumpectomy alone) in the single‐incision group.^5^

Hypertrophic scarring of the axilla following SLNB or AD has been reported in the literature to be 17%.^15^, ^16^ Obviously a single‐incision technique negates this which may be of particular importance for patients of Black and Asian ethnicity, who have been reported to have up to 20 times the rates of keloid scarring.^16^, ^17^ Additionally, this technique avoids a medial chest wall scar in the cleavage triangle during IMN dissection, a location prone to keloid scarring.

Scar pain following axillary surgery has been reported to be 3.7% (SLNB) and 13.7% (AD) which may be mitigated by avoiding an additional axillary skin incision.^15^, ^16^ This was corroborated by Nguyen‐Strauli *et al*.^11^ where patients reported axillary pain at 1.2% with a single‐incision technique compared to 10.4% with two incisions (*P* = 0.01) at three or more months follow‐up post‐operatively.

While technically the axilla is most accessible via incisions designed for tumours in the upper outer quadrant, use of a single incision has been demonstrated to be feasible in tumours of all areas of the breast accessed from a wide range of incisions, including both level one and two oncoplastic techniques.^1^, ^2^, ^5^, ^6^, ^9^, ^10^, ^11^, ^12^ As outlined in all the studies describing the technique in detail, appropriate case selection, use of retromammary plane for dissection, with long retractors, appropriate lighting and utilizing the ‘moving breast window’ is critical to success.^1^, ^2^, ^6^

The technique should not compromise the best oncological and cosmetic breast incision. While it can be considered for all breast cancers, each case should be carefully assessed so as not to compromise the safety and accuracy of the sentinel lymph node procedure with oncological principles as the primary priority. Considerations should include the presence of implants, medial tumours, small dense breasts with no ptosis and pectus excavatum, all of which were reasons for the failure of this technique.^1^, ^5^

While none of the studies have had sufficient sample size or follow‐up to adequately assess lymphoedema, Nguyen‐Strauli *et al*.^11^ reported two (4.2%) incidences of lymphoedema in their two‐incision arm compared with no cases for the single incision arm *P* = 0.056. It is postulated that leaving the skin intact in the axilla would spare a small number of dermal lymphatics as well as facilitate early mobilization with decreased pain. Whether a true difference in lymphoedema rates between the two approaches remains unanswered.

The average sentinel nodal harvest of 2–4 among all studies included in this review is comparable with most large trials which average between 2 and 3 nodes harvested.^18^ However, despite an equivalent nodal harvest and margin rate, the current body of literature lacks sufficient data on the oncological outcomes of this technique. Comprehensive, larger‐scale comparative studies with longer‐term follow‐up is necessary to confirm oncological non‐inferiority. Nevertheless, the single‐incision technique has been demonstrated in the literature to be a surgically safe, efficient and cosmetically advantageous approach with improved axillary pain and patient satisfaction.

## Conclusion

WLE and SLNB through a single incision is a surgically safe and feasible procedure. In appropriately selected cases it can be considered in all quadrants of the breast with superior cosmetic outcomes, decreased pain and improved patient satisfaction. Further research and long‐term follow‐up studies are needed to consolidate these findings and confirm oncological safety.

## Conflict of interest

Andrew J. Spillane is an Editorial Board member of ANZ Journal of Surgery and a co‐author of this article. To minimize bias, they were excluded from all editorial decision‐making related to the acceptance of this article for publication. Otherwise, Lucy P. Aitchison and Andrew J. Spillane have no conflicts of interest or financial arrangements to disclose.

## Author contributions

**Lucy P. Aitchison:** Data curation; formal analysis; investigation; methodology; writing – original draft; writing – review and editing. **Andrew J. Spillane:** Conceptualization; supervision; writing – review and editing.

## Supporting information


**Data S1** Supporting information.

## References

[1] Spillane AJ , Brennan ME . Minimal access breast surgery: a single breast incision for breast conservation surgery and sentinel lymph node biopsy. Eur. J. Surg. Oncol. 2009; 35: 380–386. 18757165 10.1016/j.ejso.2008.07.009

[2] Cocilovo C , Boolbol SK , Valdes E , Feldman S . Less is more: transmammary axillary lymph node evaluation: an initial clinical experience. Am. J. Surg. 2006; 192: 478–480. 16978953 10.1016/j.amjsurg.2006.06.025

[3] Smellie WJ , Yeatman M , Nash AG . A single axillary crease incision for wide local excision and axillary clearance in breast cancer. Ann. R. Coll. Surg. Engl. 1994; 76: 197–199. 8017816 PMC2502312

[4] Clough KB , Soussaline M , Campana F , Salmon RJ . Mammoplasty combined with irradiation: conservative treatment of breast cancer localized in the lower quadrant. Ann. Chir. Plast. Esthet. 1990; 35: 117–122. 1696083

[5] Acea‐Nebril B , García‐Novoa A , Cereijo‐Garea C , Builes‐Ramirez S , Bouzon‐Alejandro A , Mosquera‐Oses J . Single‐incision approach for breast‐conserving surgery: effectiveness, complications and quality of life. Ann. Surg. Oncol. 2019; 26: 2466–2474. 31102095 10.1245/s10434-019-07443-3

[6] Bromberg SE , Giordano R . Prime incision and modified moving window: a minimally invasive access for breast cancer surgical treatment. World J. Plast. Surg. 2016; 5: 252–258. 27853688 PMC5109386

[7] Noguchi M , Inokuchi M . Moving window operation for breast‐conserving surgery. Breast Cancer 2010; 17: 56–60. 19387777 10.1007/s12282-009-0104-1

[8] Page MJ , McKenzie JE , Bossuyt PM *et al*. Statement: an updated guideline for reporting systematic reviews. BMJ 2020; 2021: 372. 10.1136/bmj.n71 PMC8005924 33782057

[9] Bromberg SE , Moraes PR , Ades F . Prime incision: a minimally invasive approach to breast cancer surgical treatment—a two‐cohort retrospective comparison with conventional breast conserving surgery. PLoS One 2018; 13: e0191056. 29346403 10.1371/journal.pone.0191056 PMC5773166

[10] Lovasik BP , Seidel RL , Novello M , Torres MA , Losken A , Rizzo M . Single incision for oncologic breast‐conserving surgery and sentinel node biopsy in early stage breast cancer: a minimally invasive approach. Breast J. 2019; 25: 41–46. 30511408 10.1111/tbj.13158

[11] Nguyen‐Sträuli BD , Frauchiger‐Heuer H , Talimi‐Schnabel J , Loesch JM , Vorburger D , Dedes KJ . Single‐incision for breast‐conserving surgery through round block technique. Surg. Oncol. 2022; 44: 101847. 36126348 10.1016/j.suronc.2022.101847

[12] Bromberg S , Figueiredo Moraes P , Tenorio do Amaral P , Bromberg D . Minimally invasive breast surgery: a single incision approach for early breast cancer treatment—a descriptive analysis of 94 cases. Oncol. Case Rep. J. 2020; 3: 1025.

[13] Mohsen SM , El Azazy M , Hamed MK . Single incision lateral mammoplasty as an oncoplastic technique in laterally located breast cancer: a prospective controlled study. Ain Shams J. Surg. 2023; 16: 195–202.

[14] Morsy MM , Abdelfattah AR , Israel JR , Zordok AAA . A single axillary crease incision for wide local excision and axillary clearance in early breast cancer. Eur. J. Mol. Clin. Med. 2022; 9: 895–902.

[15] Langer I , Guller U , Berclaz G *et al*. Morbidity of sentinel lymph node biopsy alone versus sentinel lymph node biopsy and completion axillary lymph node dissection after breast cancer surgery: a prospective Swiss multicenter study on 659 patients. Ann. Surg. 2007; 245: 452–461. 17435553 10.1097/01.sla.0000245472.47748.ec PMC1877006

[16] Smith JC , Opalenik SR , Williams SM , Russell SB . Gene profiling of keloid fibroblasts shows altered expression in multiple fibrosis‐associated pathways. J. Invest. Dermatol. 2008; 128: 1298–1310. 17989729 10.1038/sj.jid.5701149 PMC2933038

[17] Swenson A , Paulus JK , Jung Y *et al*. Natural history of keloids: a sociodemographic analysis using structured and unstructured data. Dermatol. Ther. 2024; 14: 131–149. 10.1007/s13555-023-01070-3 PMC10828254 38066233

[18] Mansel RE , Fallowfield L , Kissin M *et al*. Randomized multicenter trial of sentinel node biopsy versus standard axillary treatment in operable breast cancer: the ALMANAC trial. J. Natl. Cancer Inst. 2006; 98: 599–609. 16670385 10.1093/jnci/djj158

