# Supplementary material for: Single breast incision for breast conservation surgery and sentinel lymph node biopsy: a systematic review
Source: ANZ J Surg. 2025 Jan 31;95(1-2):41–6. doi: 10.1111/ans.19389 (PMC11874896; doi:10.1111/ans.19389)
Supplement: Supplementary file 1 — Data S1 Supporting information. [file ANS-95-41-s001.docx]

**Supplementary Material:**

**Table 2: Distribution of tumours**

| **Study first author (year published)** | **Upper outer quadrant (%)** | **Upper inner quadrant (%)** | **Lower outer quadrant (%)** | **Lower inner quadrant (%)** | **Retroareolar or central (%)** | **Lateral (%)** | **Medial (%)** | **Upper (%)** | **Lower (%)** |
| --- | --- | --- | --- | --- | --- | --- | --- | --- | --- |
| Cocilovo (2006) | 47.7 | 11.3 | 9.1 | 0 | 31.8 | 0 | 0 | 0 | 0 |
| Spillane (2009) | 33 | 11 | 15 | 6 | 3 | 9 | 6 | 12 | 5 |
| Bromberg (2016) | 34.8 | 17.4 | 8.7 | 8.7 | 0 | 8.7 | 4.35 | 13 | 4.35 |
| Bromberg (2018) | 47 | 12 | 26 | 12 | 3 | 0 | 0 | 0 | 0 |
| Lovasik (2018) | 85.4 | 8 | 2 | 2 | 2 | 0 | 0 | 0 | 0 |
| Acea-Nebril (2019) | 64.47 | 22.97 | 11.18 | 5.26 | 6.58 | 0 | 0 | 0 | 0 |
| Bromberg (2020) | 31.4 | 8 | 5.7 | 4.5 | 8 | 21.6 | 2.3 | 8 | 8 |
| Nguyen-Strauli (2022) | 47.7 | 27.9 | 17.4 | 3.5 | 3.5 | 0 | 0 | 0 | 0 |
| Morsy (2022) | 100 | 0 | 0 | 0 | 0 | 0 | 0 | 0 | 0 |
| Mohsen (2023) | 100 | 0 | 0 | 0 | 0 | 0 | 0 | 0 | 0 |

**Appendix: (Search outcomes)**

| Pubmed | ((breast conserving surgery OR wide local excision OR lumpectomy OR tumourectomy OR tumorectomy) AND (sentinel lymph node biopsy OR axilla* OR internal mammary)) AND (single incision OR (minimal access breast surgery OR transmammary axillary lymph node evaluation OR minimally invasive breast surgery)) | 115 |
| --- | --- | --- |
| Embase | Embase Classic <1947 to 1973> Part 1 of 2  Embase <1974 to 2024 July 19>  1 ((breast conserving surgery or wide local excision or lumpectomy or tumourectomy or tumorectomy) and (sentinel lymph node biopsy or axilla* or internal mammary) and (single incision or (minimal access breast surgery or transmammary axillary lymph node evaluation or minimally invasive breast surgery))).mp. [mp=title, abstract, heading word, drug trade name, original title, device manufacturer, drug manufacturer, device trade name, keyword heading word, floating subheading word, candidate term word] 24  2 (breast conserving surgery or wide local excision or lumpectomy or tumourectomy or tumorectomy).mp. [mp=title, abstract, heading word, drug trade name, original title, device manufacturer, drug manufacturer, device trade name, keyword heading word, floating subheading word, candidate term word] 30204  3 (sentinel lymph node biopsy or axilla* or internal mammary).mp. [mp=title, abstract, heading word, drug trade name, original title, device manufacturer, drug manufacturer, device trade name, keyword heading word, floating subheading word, candidate term word] 120640  4 (single incision or minimal access breast surgery or transmammary axillary lymph node evaluation or minimally invasive breast surgery).mp. [mp=title, abstract, heading word, drug trade name, original title, device manufacturer, drug manufacturer, device trade name, keyword heading word, floating subheading word, candidate term word] 7309  5 2 and 3 and 4 24  6 2 and 4 28 | 28 |
| Medline | Ovid MEDLINE(R) ALL <1946 to July 19, 2024>  1 (breast conserving surgery or wide local excision or lumpectomy or tumourectomy or tumorectomy).mp. [mp=title, book title, abstract, original title, name of substance word, subject heading word, floating sub-heading word, keyword heading word, organism supplementary concept word, protocol supplementary concept word, rare disease supplementary concept word, unique identifier, synonyms, population supplementary concept word, anatomy supplementary concept word] 14964  2 (sentinel lymph node biopsy or axilla* or internal mammary).mp. [mp=title, book title, abstract, original title, name of substance word, subject heading word, floating sub-heading word, keyword heading word, organism supplementary concept word, protocol supplementary concept word, rare disease supplementary concept word, unique identifier, synonyms, population supplementary concept word, anatomy supplementary concept word] 71185  3 (single incision or minimal access breast surgery or transmammary axillary lymph node evaluation or minimally invasive breast surgery).mp. [mp=title, book title, abstract, original title, name of substance word, subject heading word, floating sub-heading word, keyword heading word, organism supplementary concept word, protocol supplementary concept word, rare disease supplementary concept word, unique identifier, synonyms, population supplementary concept word, anatomy supplementary concept word] 4094  4 1 and 2 and 3 13  5 from 4 keep 1-13 13 | 13 |
| GoogleScholar | "breast surgery" minimally OR invasive "single incision" -robotic -endoscopic | 270 |
